# Public Attitudes and Factors of COVID-19 Testing Hesitancy in the United Kingdom and China: Comparative Infodemiology Study

**DOI:** 10.2196/26895

**Published:** 2021-08-27

**Authors:** Leesa Lin, Yi Song, Qian Wang, Jialu Pu, Fiona Yueqian Sun, Yixuan Zhang, Xinyu Zhou, Heidi J Larson, Zhiyuan Hou

**Affiliations:** 1 Department of Infectious Disease Epidemiology London School of Hygiene & Tropical Medicine London United Kingdom; 2 Laboratory of Data Discovery for Health Hong Kong Science Park Hong Kong SAR China; 3 School of Public Health Fudan University Shanghai China; 4 NHC Key Laboratory of Health Technology Assessment Fudan University Shanghai China; 5 Global Health Institute Fudan University Shanghai China

**Keywords:** COVID-19, test, public response, sentiment, social listening, United Kingdom, China

## Abstract

**Background:**

Massive community-wide testing has become the cornerstone of management strategies for the COVID-19 pandemic.

**Objective:**

This study was a comparative analysis between the United Kingdom and China, which aimed to assess public attitudes and uptake regarding COVID-19 testing, with a focus on factors of COVID-19 testing hesitancy, including effectiveness, access, risk perception, and communication.

**Methods:**

We collected and manually coded 3856 UK tweets and 9299 Chinese Sina Weibo posts mentioning COVID-19 testing from June 1 to July 15, 2020. Adapted from the World Health Organization’s 3C Model of Vaccine Hesitancy, we employed social listening analysis examining key factors of COVID-19 testing hesitancy (confidence, complacency, convenience, and communication). Descriptive analysis, time trends, geographical mapping, and chi-squared tests were performed to assess the temporal, spatial, and sociodemographic characteristics that determine the difference in attitudes or uptake of COVID-19 tests.

**Results:**

The UK tweets demonstrated a higher percentage of support toward COVID-19 testing than the posts from China. There were much wider reports of public uptake of COVID-19 tests in mainland China than in the United Kingdom; however, uncomfortable experiences and logistical barriers to testing were more expressed in China. The driving forces for undergoing COVID-19 testing were personal health needs, community-wide testing, and mandatory testing policies for travel, with major differences in the ranking order between the two countries. Rumors and information inquiries about COVID-19 testing were also identified.

**Conclusions:**

Public attitudes and acceptance toward COVID-19 testing constantly evolve with local epidemic situations. Policies and information campaigns that emphasize the importance of timely testing and rapid communication responses to inquiries and rumors, and provide a supportive environment for accessing tests are key to tackling COVID-19 testing hesitancy and increasing uptake.

## Introduction

As the number of COVID-19 cases accelerated globally in early 2020, many public health experts advocated for widespread rapid testing that could complement other containment strategies, such as hand washing, contact tracing, and quarantine, and that should be viewed as important as face covering, social distancing, and vaccines [1,2]. There are two widely accepted types of tests as follows: (1) a nucleic acid test, which is a polymerase chain reaction test that detects RNA (or genetic material) specific to the virus, and (2) an antigen test, which is a rapid turnaround virus test from a lateral flow device that can process COVID-19 samples on site without the need for laboratory equipment. Community-wide COVID-19 testing helped public health investigators understand the prevalence, contagiousness, and mortality of the disease [3], and has made it possible for communities to exit lockdowns and rapidly control potential resurgences while awaiting a safe vaccine. China, Singapore, Germany, and South Korea have been among the most early and aggressive countries in utilizing widespread frequent rapid tests (offered freely to residents) as a central pillar of their multipronged epidemic control strategies. In China, mass testing has been employed as a standard procedure in places where new outbreaks of COVID-19 surged [4]. During the resurgence in Beijing in June 2020, 3.56 million individuals at risk were tracked and tested [5], and the outbreak was quickly brought under control. In contrast, countries, such as the United Kingdom and Japan, had delays in rolling out mass testing [6], and asymptomatic individuals and high-risk populations (ie, health care workers) were not able to access testing in the pandemic’s early stage due to limited capacity [7]. Two and a half months after nationwide lockdown, on May 28, 2020, the United Kingdom eventually launched the National Health System (NHS) Test and Trace program, a “world-beating system” that the Prime Minister had pledged to deliver as a central part of the government’s COVID-19 recovery strategy. By July, people with COVID-19 symptoms could receive a test from the NHS without charge, and those engaged in high-risk jobs were promised regular testing by the UK government [8,9]. However, since it was introduced, the program has been repeatedly criticized for not meeting expectations. As the pandemic response progresses, the challenge of conducting COVID-19 mass testing will transition from inadequate testing capacity to inadequate uptake [10] due to pandemic fatigue, test anxiety, stigmatization, rumors, misinformation, fear of isolation and quarantine, and other disincentives.

Infodemiology, first introduced by Dr Gunther Eysenbach in early 2000 as the epidemiology of (mis)information [11], is an emerging field of research on the distribution and determinants of user-contributed health information and misinformation across the internet or in a population, with the ultimate aim of improving public health and public policy [12]. The COVID-19 pandemic created a paradigm shift in communication and infodemiology, as widespread negative health and socioeconomical impacts were observed to be caused by two concurrent pandemics (the novel coronavirus and misinformation). Social listening in the context of public health has been found to be an effective tool that offers real-time big data on public sentiment and opinions for informing and assessing governments’ risk communication strategies and public reactions, especially during acute epidemic outbreaks, such as the 2009/2010 H1N1 [13], 2013/2014 Middle East respiratory syndrome (MERS) [14], and 2014 Ebola [13] epidemics. Unlike traditional research methods (eg, surveys or in-depth interviews), where opinion gathering is limited to interactions between researchers and participants, social listening allows for a rapid and thorough scanning of a multilevel dynamic information environment for digital opinions derived from public contributions, interactions, and interinfluences without researcher involvement. Social listening investigates public understanding and experiences of an event (ie, risks and countermeasures), which, as depicted in Stuart Hall’s audience reception theory [15], are shaped by their individual sociocultural backgrounds and life experiences.

At-risk individuals refusing or avoiding testing could undermine a community’s epidemic control and reopening strategies. Public health experts and decision makers must monitor public sentiment and acceptance toward testing and understand the root causes of testing hesitancy. To date, research has mainly focused on COVID-19 vaccines [16] and other nonpharmaceutical measures, such as lockdowns, social distancing, and mask wearing [17-20], leaving COVID-19 testing hesitancy and avoidance underinvestigated. The United Kingdom and China have highly active microblog users and have experienced initial COVID-19 outbreaks, lockdowns, and resurgence, yet mass testing was introduced in these two countries at different stages of response. As such, this infodemiology study aimed to assess public attitudes and uptakes of COVID-19 testing in the United Kingdom and China, with a focus on the factors of testing hesitancy, including effectiveness, access, risk perception, and communication.

## Methods

### Data Collection

We collected microblog posts from popular social media platforms in the United Kingdom and China. We assessed Twitter tweets (the United Kingdom) and Sina Weibo posts (mainland China) mentioning COVID-19 testing from June 1 to July 15, 2020, after the launch of the NHS Test and Trace system in the United Kingdom and the mass testing campaign in Beijing, China, during COVID-19’s resurgence and before another resurgence in Xinjiang Autonomous Region, China. We used the Meltwater platform [21] to collect Twitter tweets and Weibo posts. The keywords used for collecting tweets or Weibo posts were “covid test,” “covid19 test,” “covid-19 test,” “coronavirus test,” “test for covid,” and “test for coronavirus” (“核酸检测” in Chinese). Overall, 59,919 tweets from the United Kingdom (including 11,249 tweets from London) and 313,092 Weibo posts from China (including 82,743 Weibo posts from Beijing) were collected with the location and time they were sent. Weibo posts were downloaded daily so as to minimize possible bias resulting from posts being removed by authorities. We also downloaded the account profile of each Weibo post, from which we extracted gender, age, and education for analysis. Only human-contributed opinions/conversations on Twitter and Weibo were included for analysis. Tweets or Weibo posts from news and organizational accounts, and tweets/posts generated by bots were identified by keyword matching, and then examined and removed by researchers. Duplicate tweets/posts, tweets/posts with identical text but from different accounts, retweets, and quotes without comments were removed. After removing posts that did not meet the inclusion criteria, we randomly sampled 10% of the tweets and posts by day for coding. In total, 3856 tweets from the United Kingdom, including 794 tweets from London, and 9299 Weibo posts from mainland China, including 3155 posts from Beijing, were included for formal analysis. Multimedia Appendix 1 shows the workflow of the inclusion and exclusion processes of the tweets and Weibo posts.

Analyses of accounts of social media users (Multimedia Appendix 2) suggested that our data of social media posts were well-representative of the entire social media user base. We found that 92.8% (3581/3856) of tweets in the United Kingdom and 97.5% (9067/9299) of Weibo posts in China were single posts sent by unique users.

To assess data representativeness, we compared Weibo users’ demographic data in our study with the “Weibo 2020 User Development Report” [22]. This report showed that active female users accounted for 54.6% of the user base and that the user base is skewed toward young users; 78% of Weibo users are under 30 years old. Not all Weibo users’ profiles were available, and in our data set with users’ profiles, 67.6% (5940/8784) of users were female and 70.6% (2705/3830) were under 30 years old. Our results showed that our demographic profiles were comparable to the overall user base profile reported by Weibo.

### Data Analysis

This study employed content analysis of social media data in relation to COVID-19 testing [23-25], complemented by contextual epidemic data. COVID-19 epidemic data from the United Kingdom and mainland China were derived for trend analysis [26,27]. We plotted the trends of daily new COVID-19 case numbers in the United Kingdom and China to describe the epidemic context where mass testing programs were introduced.

We identified and classified social media posts that expressed personal opinions/discussions on COVID-19 testing. Public attitudes toward COVID-19 testing were manually screened and coded based on the three different positions one might take as follows: dominant (understanding and accepting the objectives of the test), negotiated (reacting with a mixture of acceptance and rejection), and oppositional (opposite to the dominant position and completely rejecting the test) [15]. To investigate the determinants of public attitudes toward COVID-19 testing, we employed a deductive approach with a coding framework that was adapted from the World Health Organization’s “3Cs” model of hesitancy toward vaccination [28], and this “3Cs” model was also applied for other public health behaviors, such as COVID-19 testing. The coding framework, presented in Multimedia Appendix 3, covers major factors of COVID-19 testing hesitancy, including *confidence* (degree of trust in the effectiveness and safety of the test), *complacency* (perceptions of personal risk associated with the disease and test), and *convenience* (influencers of the decision to get the test, eg, availability, affordability, and geographical accessibility), as well as *communication* (information inquiries and rumors about COVID-19 testing) [29].

In execution, we first developed a codebook with code definitions. Then, two researchers (YS and QW) coded a subsample of 500 posts independently, and when appropriate, refined the codebook. When necessary, SentiWordNet [30] was referenced. Using the final codebook, another subsample of 200 posts was independently coded to check the intercoder reliability. Cohen κ [31] was used to measure intercoder reliability, which reached κ=0.825 after the final revision. Lastly, during the formal coding phase, four coders were trained and divided into two pairs of coders (YS and JP, and QW and YZ). Each pair independently coded a subset of tweets/posts, with a third coder (QW or YS) checking and resolving any disagreements.

A descriptive analysis was performed to show the percentage of topics for both Twitter and Weibo data. The time trends were plotted for percentages of tweets or Weibo posts with various attitudes toward general COVID-19 tests by week. Geographical distributions of post numbers and percentages of oppositional attitudes across the United Kingdom and mainland China were plotted by regions or provinces. The chi-square test was used to determine differences in attitudes or behaviors toward COVID-19 by gender, age, and education.

## Results

### Epidemic Context: Daily New COVID-19 Case Numbers in the United Kingdom and China

**Figure 1:**
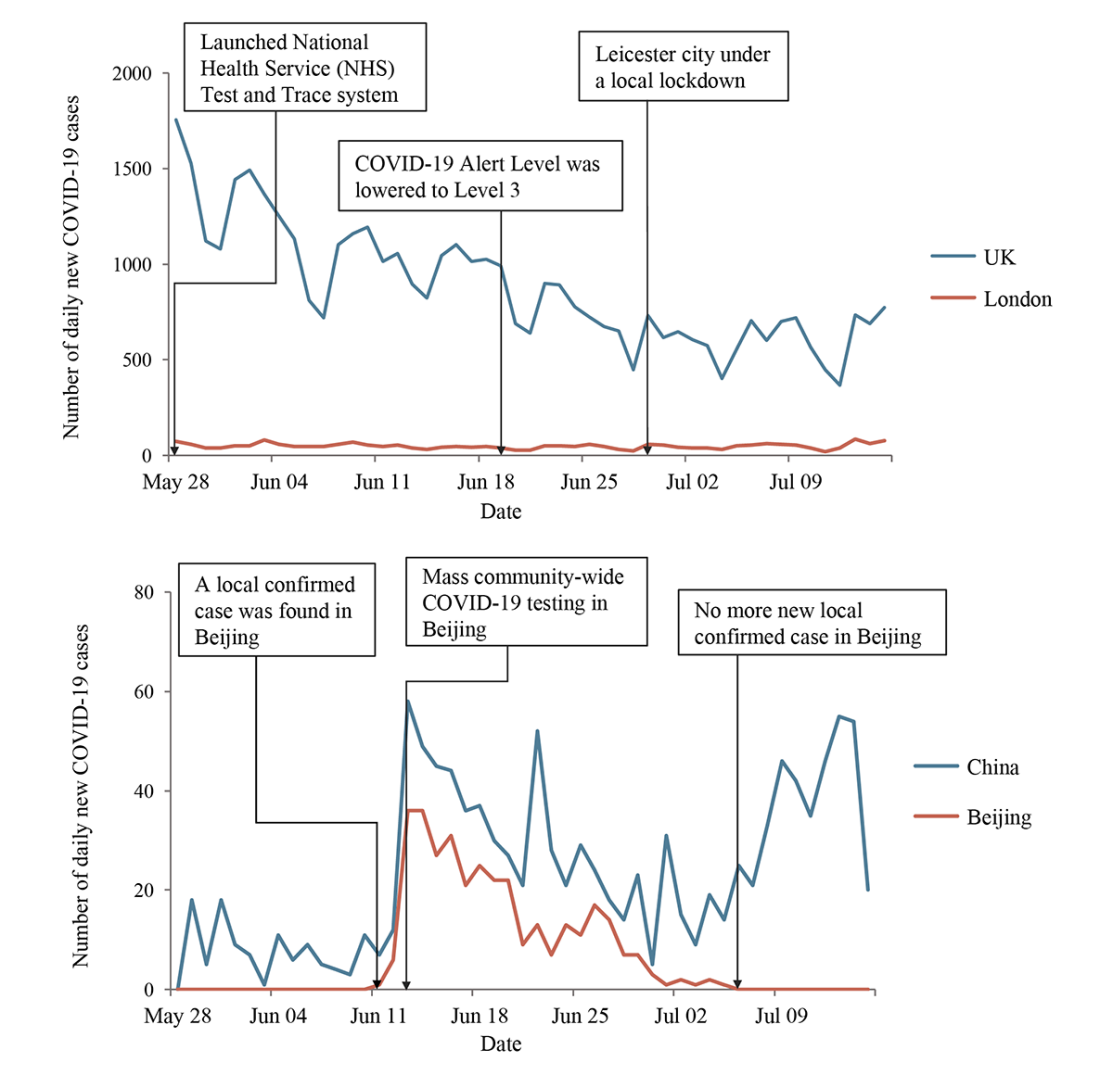
Numbers of daily new COVID-19 cases in the United Kingdom (UK), London, mainland China, and Beijing from June 1 to July 15, 2020 [26,27].

From June 1 to July 15, 2020, daily new COVID-19 confirmed cases demonstrated a decreasing trend in the United Kingdom, stabilizing at around 50 in London (Figure 1). With the decrease in new cases, the COVID-19 Alert Level in the United Kingdom was downgraded from level 4 to level 3 on June 19, representing that the COVID-19 epidemic was in general circulation with a demonstrable reduction in the number of cases and deaths [32]. On June 29, Leicester became the first city in the United Kingdom to undergo a local lockdown after a resurgence of cases. Concurrently, daily new cases in China fluctuated under 60, with the majority being in Beijing. There were no more local confirmed cases in Beijing after July 6.

### Social Listening: Public Attitudes Toward COVID-19 Testing

#### Overall Analysis

**Figure 2:**
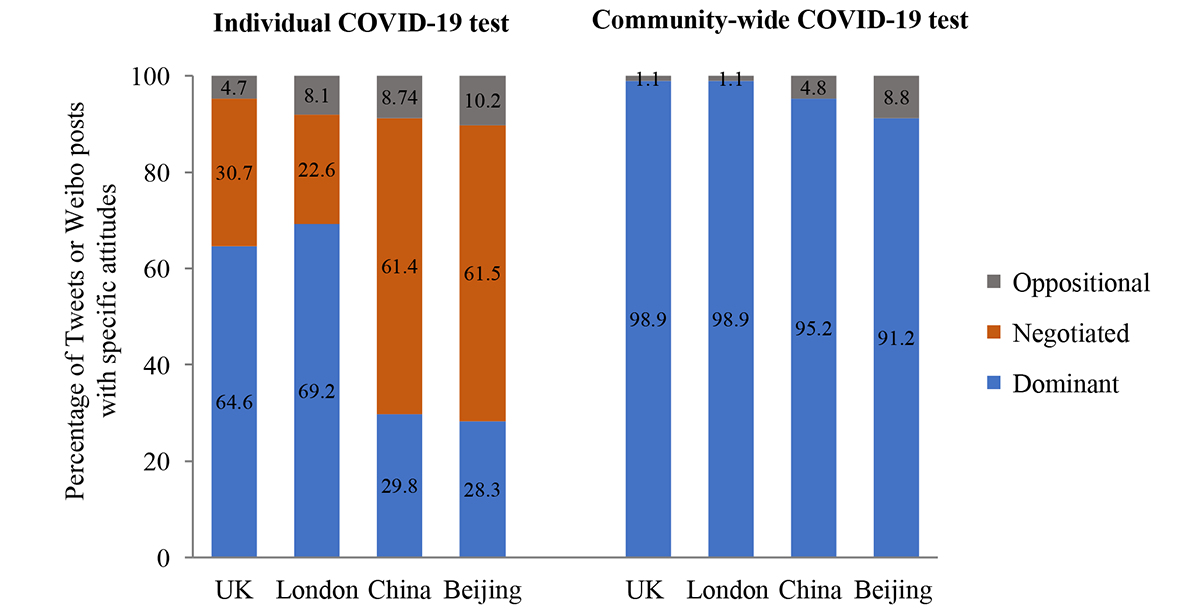
Percentage of tweets or Weibo posts with attitudes toward individual COVID-19 tests and community-wide tests from June 1 to July 15, 2020. UK: United Kingdom.

In the United Kingdom, 64.6% (2390/3700) of tweets across the country and 69.2% (520/751) from London showed dominant views on individual COVID-19 tests in general. Moreover, 30.7% (1136/3700) of tweets from the United Kingdom and 22.6% (170/751) from London showed negotiated views on individual tests, while 4.7% (174/3700) across the country and 8.1% (61/751) from London opposed it (Figure 2). In China, about 30% of posts (country wide: 2649/8879, 29.8%; Beijing: 848/2991, 28.3%) showed dominant views on COVID-19 tests in general. Moreover, over 60% of posts (country wide: 5454/8879, 61.4%; Beijing: 1839/2991, 61.5%) showed negotiated views, and 10% or less (country wide: 776/8879, 8.7%; Beijing: 304/2991, 10.2%) opposed it. For example, tweets/posts with dominant views on individual COVID-19 tests included “*need larger testing capacity and faster results*,” negotiated tweets/posts included “*does one need to have a covid-19 test before travelling to the US*,” and oppositional tweets/posts included “*30% of negative coronavirus tests are wrong.*” Individuals in the United Kingdom (23/2075, 1.1%) and London (6/530, 1.1%) showed less opposition to government-led community-wide mass COVID-19 testing than did those in mainland China (76/1594, 4.8%) and Beijing (58/661, 8.8%). For example, tweets/posts with dominant views on community-wide mass COVID-19 testing included “*support NHS and care workers routine weekly COVID-19 tests*” and oppositional tweets/posts included “*why would people with no symptoms take a test that tells them they're sick*.” A total of 2487 tweets from the United Kingdom expressed discontent with governmental COVID-19 testing practices, including taxing tests, voting against routine testing for front-line workers, publishing wrong or untimely data, and other complaints. In China, 304 Weibo posts questioned the necessity of having to obtain test results before travelling, visiting doctors, etc.

#### Time Trend Analysis and Geographical Mapping

**Figure 3:**
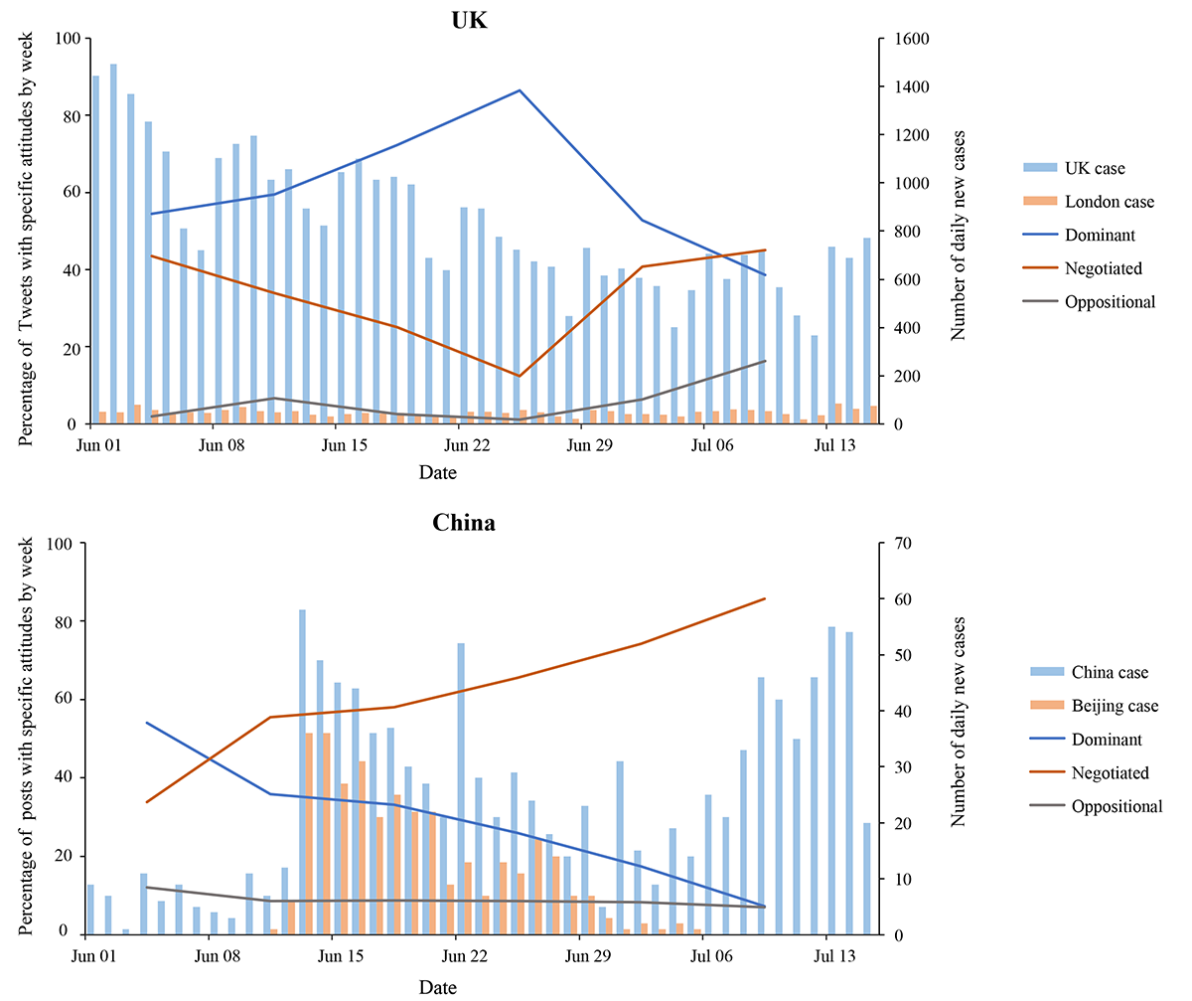
Percentage of tweets or Weibo posts with attitudes toward COVID-19 testing by week and daily new cases from June 1 to July 15, 2020. UK: United Kingdom.

**Figure 4:**
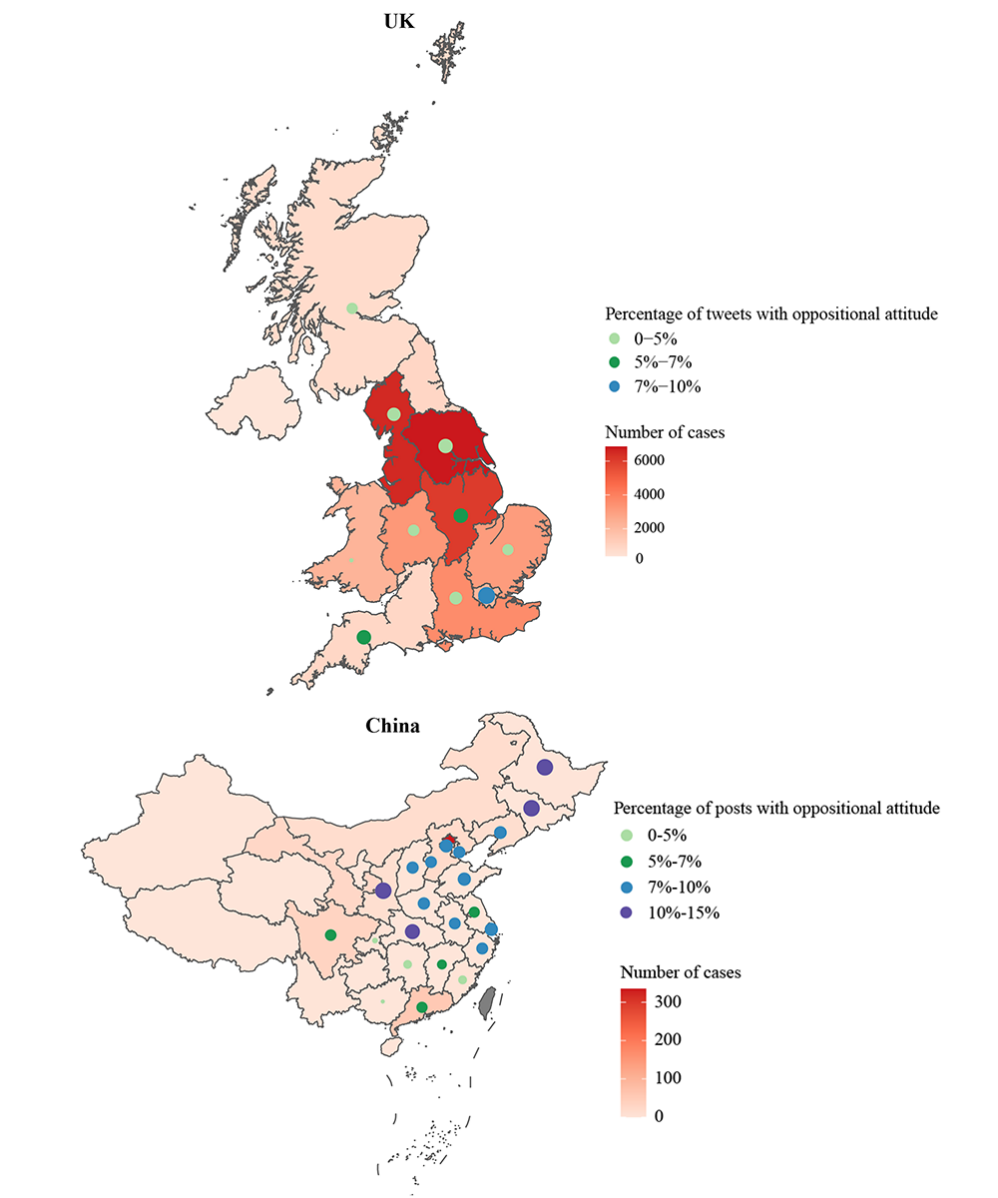
Percentage of tweets/Weibo posts with oppositional attitude toward COVID-19 testing and number of cases by geographical distribution in the United Kingdom (UK) and mainland China from June 1 to July 15, 2020 (regions with less than 50 tweets/posts are not shown).

Time trend analysis (Figure 3) showed that, in the United Kingdom, posts with dominant attitudes toward individual COVID-19 tests first increased from 54.5% (533/978) to 86.5% (1066/1233) and then dropped to 38.6% (180/466) after the enactment of The Health Protection (Coronavirus) Regulations in July 2020 [33], while tweets with oppositional attitudes increased from 1.9% (19/978) to 16.3% (76/466). In China, Weibo posts with dominant attitudes reduced from 54.1% (380/703) to 7.3% (30/412) during this period, while those with negotiated attitudes increased from 33.9% (238/703) to 85.7% (353/412) and posts with oppositional attitudes slightly dropped from 12.1% (85/703) to 7.0% (29/412). Regional analyses (Figure 4) showed that the percentage of tweets/posts in opposition to testing generally corresponded with low cases in their respective regions, with the exceptions of London (64/852, 7.5%) and the East Midlands (8/157, 5.1%). Oppositional tweets mostly worried about false negative testing results and that someone could get infected after testing negative, leading to more cases. Weibo posts from Beijing showed a slightly higher level of oppositional attitudes than London (304/3155, 9.6%), mostly questioning the cost-effectiveness of implementing mass testing when daily new cases in China fluctuated under 60.

### Self-Reported Uptake of COVID-19 Tests

Overall, 4.6% (178/3856) of tweets across the United Kingdom and 4.9% (39/794) from London reported intending to undergo or having undergone COVID-19 tests (Table 1). In the United Kingdom, driving forces for undergoing testing included personal health needs due to possible exposure, symptoms, or worry (37/86, 43%), mandatory testing policies for travel (30/86, 35%), and mass community-wide testing led by the government (19/86, 22%). Comparatively, more Weibo users reported having undergone COVID-19 testing in China (3318/9299, 35.7%) and Beijing (1462/3155, 46.3%). A total of 1600 Weibo posts (1600/9299, 17.2%) from China reported driving forces for undergoing testing, including community-wide testing led by the government (784/1600, 49.0%), mandatory testing policies for travel (659/1600, 41.2%), and personal health needs (163/1600, 10.2%). Government-led community-wide testing was reported to be the main driving force for undergoing testing in Beijing (543/824, 65.9%).

**Table 1:** Uptake of COVID-19 tests in the United Kingdom and mainland China.

Uptake and driving forces			United Kingdom (N=3856), n (%)		London (N=794), n (%)		China (N=9299), n (%)		Beijing (N=3155), n (%)
**Self-reported uptake of COVID-19 tests**			178 (4.6)		39 (4.9)		3318 (35.7)		1462 (46.3)
	Plan to take a test	35 (0.9)		7 (0.9)		811 (8.7)		417 (13.2)	
	Have taken a test	143 (3.7)		32 (4.0)		2507 (27.0)		1045 (33.1)	
**Driving force for taking a COVID-19 test**			86 (2.2)		25 (3.2)		1600 (17.2)		824 (26.1)
	Personal health needs	37 (1.0)		14 (1.8)		163 (1.8)		64 (2.0)	
	Mandatory testing policies for travel	30 (0.8)		9 (1.1)		659 (7.1)		223 (7.1)	
	Community-wide testing by governments	19 (0.5)		2 (0.3)		784 (8.4)		543 (17.2)	
Others taking COVID-19 tests			86 (2.2)		16 (2.0)		277 (3.0)		102 (3.2)

### Major Factors of COVID-19 Testing Hesitancy

#### Convenience: Access to and Experience With COVID-19 Tests

In the United Kingdom, 1.1% (43/3856) of tweets shared their experiences of undergoing COVID-19 tests, of which, 69.8% (30/43) reported being uncomfortable, 16.3% (7/43) reported being nervous, and 14.0% (6/43) reported no discomfort (Table 2). Comparatively, more Weibo posts shared the overall experience of taking a COVID-19 test from China (753/9299, 8.1%) and Beijing (221/3155, 7.0%). Furthermore, 9.2% (356/3856) of tweets in the United Kingdom and 16.6% (132/794) of tweets in London discussed the logistical process of obtaining a test, including access to an appointment, wait time to undergo testing, including queues and heatstroke while waiting, wait time for test results, and others. Discussions about the logistical process of obtaining a test in China (2005/9299, 21.6%) and Beijing (754/3155, 23.9%) mostly about the wait time to undergo testing received significant attention (China: 1579/2005, 78.8%; Beijing: 557/754, 73.9%).

The price of COVID-19 tests was mostly mentioned in London (53/794, 6.7%) across the United Kingdom (96/3856, 2.5%). These discussions included calls for free testing and dissatisfaction with high expenses, complaints about self-paying without reimbursement by medical insurance, and satisfaction with free-of-charge testing when available. In China, 6.4% (598/9299) of posts across the country and 6.2% (196/3155) from Beijing discussed the price of COVID-19 tests. The discussions included satisfaction with free-of-charge community-wide testing by governments and different attitudes toward self-paying prices, being either reasonable or burdensome. Regarding priority groups for COVID-19 testing, tweeters in the United Kingdom mentioned support for weekly testing for NHS staff and care staff and calling for priority testing to be extended. In China, posts concerning priority groups for COVID-19 testing referred to support for people with possible risk exposure, taxi drivers, and couriers to receive priority testing.

**Table 2:** Convenience of COVID-19 tests in the United Kingdom and mainland China.

Convenience of COVID-19 tests			United Kingdom (N=3856), n (%)		London (N=794), n (%)		China (N=9299), n (%)		Beijing (N=3155), n (%)
**Experience of taking a COVID-19 test**			43 (1.1)		8 (1.0)		753 (8.1)		221 (7.0)
	Feel uncomfortable	30 (0.8)		5 (0.6)		476 (5.1)		118 (3.7)	
	Feel nervous	7 (0.2)		2 (0.3)		234 (2.5)		70 (2.2)	
	Do not feel uncomfortable	6 (0.2)		1 (0.1)		111 (1.2)		43 (1.4)	
**Logistical process of obtaining a COVID-19 test**			356 (9.2)		132 (16.6)		2005 (21.6)		754 (23.9)
	Access to an appointment	25 (0.6)		8 (1.0)		93 (1.0)		52 (1.6)	
	Wait time to take a test	53 (1.4)		19 (2.4)		1579 (17.0)		557 (17.7)	
	Wait time for the test result	82 (2.1)		10 (1.3)		174 (1.9)		114 (3.6)	
	Others	211 (5.5)		99 (12.5)		186 (2.0)		48 (1.5)	
Tribute to medical staff			69 (1.8)		15 (1.9)		1914 (20.6)		480 (15.2)
Price of COVID-19 testing			96 (2.5)		53 (6.7)		598 (6.4)		196 (6.2)
Priority groups for COVID-19 testing			665 (17.2)		9 (1.1)		271 (2.9)		101 (3.2)

#### Confidence and Complacency Toward COVID-19 Tests

In both the United Kingdom and China, social media users expressed a high perceived risk of the COVID-19 pandemic (United Kingdom: 139/152, 91.4%; China: 789/813, 97.0%) (Table 3). Moreover, 4.3% (164/3856) of tweets across the United Kingdom and 6.2% (49/794) from London concerned the effectiveness of COVID-19 tests, and of them, 20.7% (34/164) across the United Kingdom and 32.7% (16/49) from London expressed confidence in its effectiveness, while 79.3% (130/164) across the United Kingdom and 67.3% (33/49) from London expressed doubts. In China, 4.5% (417/9299) of posts across the country and 5.2% (164/3155) from Beijing concerned test effectiveness, and of them, 65.9% (275/417) across China and 62.2% (102/164) from Beijing expressed confidence in its effectiveness, while 34.1% (142/417) across China and 37.8% (62/164) from Beijing expressed doubts.

**Table 3:** Confidence and complacency toward COVID-19 tests in the United Kingdom and mainland China.

Confidence and complacency				United Kingdom (N=3856), n (%)		London (N=794), n (%)		China (N=9299), n (%)		Beijing (N=3155), n (%)
**Confidence: trust in COVID-19 tests**										
	**Concern on the effectiveness of COVID-19 tests**		164 (4.3)		49 (6.2)		417 (4.5)		164 (5.2)	
		Trust tests to be effective	34 (0.9)		16 (2.0)		275 (3.0)		102 (3.2)	
		Doubt the effectiveness of tests	130 (3.4)		33 (4.2)		142 (1.5)		62 (2.0)	
	Expiration date of COVID-19 tests		1 (0.03)		0 (0)		87 (0.9)		42 (1.3)	
	Incidental risks due to COVID-19 tests		14 (0.4)		3 (0.4)		206 (2.2)		113 (3.6)	
**Complacency: perception of COVID-19 risk**										
	**Perception of COVID-19 risk**		152 (3.9)		45 (5.7)		813 (8.7)		299 (9.5)	
		High risk	139 (3.6)		44 (5.5)		789 (8.5)		286 (9.1)	
		Low risk	13 (0.3)		1 (0.1)		24 (0.3)		13 (0.4)	

#### Communication: Information Inquiries and Rumors Related to COVID-19 Tests

Information inquiries and rumors about COVID-19 tests (Table 4) could be found on both UK and Chinese social media platforms (United Kingdom: 75/3856, 1.9% and 55/3856, 1.4%; China: 517/9299, 5.6% and 192/9299, 2.1%, respectively). The main information inquiries (22/75, 29.3%) mentioned in the United Kingdom about COVID-19 testing were “*how many people have received a test*” and “*delay in sharing testing data with English councils*,” while in China, 48.2% (249/517) of information inquiries were “*whether tests are needed before travelling somewhere*” and “*how much it costs to take a test*.” Concerns included the duration (expiration) of test results and incidental risks associated with COVID-19 testing, such as cross-infection and threat of asymptomatic infection from crowd gathering. Posts mentioning unproven expositions about or interpretations of COVID-19 testing–related news, events, or problems were labelled as rumors (including fake news and misinformation)*.* For example, rumors in the United Kingdom included “*COVID-19 test results were falsified*” and fake news included “*4 Tory MPs voted against weekly COVID-19 tests for NHS and care staff*.” Comparatively, rumors in China included “*medical staff earned money by COVID-19 tests*” and fake news included “*positive testing results for [person name] in [place]*.”

**Table 4:** Communication around COVID-19 tests in the United Kingdom and mainland China.

Communication around COVID-19 tests	United Kingdom (N=3856), n (%)	London (N=794), n (%)	China (N=9299), n (%)	Beijing (N=3155), n (%)
Information inquiries about COVID-19 tests	75 (1.9)	21 (2.6)	517 (5.6)	137 (4.3)
Rumors about COVID-19 tests	55 (1.4)	12 (1.5)	192 (2.1)	27 (0.9)

### Attitude and Uptake of COVID-19 Tests by the Characteristics of Social Media Posts

Tables 5 and 6 show the univariate analysis of the attitude and uptake of COVID-19 tests across sociodemographic characteristics using Weibo data from China. Male Weibo users and those over 30 years old were more likely to have a positive attitude toward individual COVID-19 testing (*P*<.001), but male users were less likely to have a positive attitude toward mass community-wide COVID-19 testing led by the government (*P*<.001) (Table 5). Additionally, females, users under 30 years old, and those with a bachelor’s degree or higher were more likely to take a COVID-19 test (*P*≤.001), and there was no significant difference in the reasons for undergoing COVID-19 testing (Table 6).

**Table 5:** Attitude toward COVID-19 tests by characteristics for Chinese Weibo posts.

Characteristic			Attitude toward individual COVID-19 tests									*P* value			Attitude toward community-wide COVID-19 tests							*P* value
			Total		Dominant, n (%)		Negotiated, n (%)		Oppositional, n (%)					Total			Dominant, n (%)		Oppositional, n (%)			
**Gender**											<.001										<.001	
	Male	2844		989 (34.8)		1587 (55.8)		268 (9.4)					660			614 (93.0)		46 (7.0)				
	Female	5940		1626 (27.4)		3814 (64.2)		500 (8.4)					910			882 (96.9)		28 (3.1)				
	Total	8784		2615 (29.8)		5401 (61.5)		768 (8.7)					1570			1496 (95.3)		74 (4.7)				
**Age (years)**											<.001										.05	
	10-30	2705		680 (25.1)		1793 (66.3)		232 (8.6)					356			344 (96.6)		12 (3.4)				
	30-90	1125		420 (37.3)		602 (53.5)		103 (9.2)					269			251 (93.3)		18 (6.7)				
	Total	3830		1100 (28.7)		2395 (62.5)		335 (8.7)					625			595 (95.2)		30 (4.8)				
**Education**											.49										.90	
	Bachelor’s degree or above	2297		704 (30.6)		1387 (60.4)		206 (9.0)					472			450 (95.3)		22 (4.7)				
	High school or below	6582		1945 (29.6)		4067 (61.8)		570 (8.7)					1122			1068 (95.2)		54 (4.8)				
	Total	8879		2649 (29.8)		5454 (61.4)		776 (8.7)					1594			1518 (95.2)		76 (4.8)				

**Table 6:** Uptake of COVID-19 tests by characteristics for Chinese Weibo posts.

Characteristic			Uptake of COVID-19 tests							*P* value		Driving force for taking a COVID-19 test								*P* value
			Total		Yes, n (%)		No, n (%)				Total			Personal health needs, n (%)		Mandatory testing policies for travel, n (%)	Community-wide tests, n (%)			
**Gender**									<.001										.44	
	Male	2844		808 (28.4)		2036 (71.6)					413		37 (9.0)		167 (40.4)		209 (50.6)			
	Female	5940		2470 (41.6)		3470 (58.4)					1170		126 (10.8)		487 (41.6)		557 (47.6)			
	Total	8784		3278 (37.3)		5506 (62.7)					1583		163 (10.3)		654 (41.3)		766 (48.4)			
**Age (years)**									<.001										.09	
	10-30	2705		1187 (43.9)		1518 (56.1)					506		60 (11.9)		229 (45.3)		217 (42.9)			
	30-90	1125		316 (28.1)		809 (71.9)					193		22 (11.4)		71 (36.8)		100 (51.8)			
	Total	3830		1503 (39.2)		2327 (60.8)					699		82 (11.7)		300 (42.9)		317 (45.4)			
**Education**									.001										.16	
	Bachelor’s degree or above	2297		922 (40.1)		1375 (59.9)					444		45 (10.1)		166 (37.4)		233 (52.5)			
	High school or below	6582		2395 (36.4)		4187 (63.6)					1162		118 (10.2)		493 (42.4)		551 (47.4)			
	Total	8879		3317 (37.4)		5562 (62.6)					1606		163 (10.1)		659 (41.0)		784 (48.8)			

## Discussion

### Principal Findings

This infodemiology study assessed public attitudes and opinions around COVID-19 testing, including both individual and government-led mass testing, by monitoring and analyzing digital conversations in the United Kingdom (Twitter) and China (Sina Weibo) with a framework of testing hesitancy (confidence, complacency, convenience, and communication). Overall, there was a higher level of support toward individual and mass COVID-19 testing in the United Kingdom and London than in mainland China and Beijing; most opposition originated from the capital cities. Time trend analyses showed that discussions about individual COVID-19 tests were mostly dominant in the United Kingdom, while Weibo posts in China showed a rise of negotiated views over testing. There were much wider reports of public uptake of COVID-19 tests in mainland China than in the United Kingdom. Personal health needs (eg, possible exposure, symptoms, and worry), mandatory testing policies for work or travel, and government-led mass testing were the main driving forces for people to undergo testing in both countries, with differences in priorities between countries. The Chinese public posted more about uncomfortable experiences and logistical barriers to testing, whereas people in the United Kingdom posted more about prices and priority groups for testing. Perceived risk of the COVID-19 disease was high in both countries. Only 5% or less of the posts discussed test effectiveness, and of them, Chinese users expressed confidence in its effectiveness, whereas British users displayed doubts. Rumors related to COVID-19 test administration and results were identified. In China, females, those under 30 years old, and those with a bachelor’s degree or higher were more likely to undergo a COVID-19 test.

Overall, discussions about COVID-19 in both countries showed low complacency (high perceived risk) for the COVID-19 disease and high confidence in testing, which translated into high levels of public support for testing. The cited driving forces for testing (personal health needs in the United Kingdom versus government-led mass testing or mandatory testing policies for travel in China) also reflected the epidemic situation and testing policies implemented in each respective context. Our data showed that, as daily new cases decreased and COVID-19 testing became routine, a negotiated position toward COVID-19 testing became the majority view, leading to an increase in acceptance and uptake behavior when needed. Epidemiologists have argued that widespread dissemination of cheap and rapid tests might be as effective as a vaccine at interrupting coronavirus transmission by identifying and isolating people with the virus when they are most infectious [1,34]. Integrating “complacency (risk perception)” of the disease and “confidence” of testing in messaging by emphasizing the importance of timely testing during an acute epidemic could increase acceptance and uptake.

“Convenience” of testing, including accessibility, frequency, and sample-to-answer time, was a popular topic of digital discussion and also one of the most important factors for effective screening, being an even higher priority than the analytical limits of detection [34]. Inquiries and rumors related to COVID-19 testing pointed to the lack of a frequent and factually correct information campaign. Furthermore, regional analysis showed an association between opposition views toward testing and low case counts, with the exceptions of London and the East Midlands, mostly because of worrying about false negative testing results and worrying that someone could get infected after testing negative, leading to more cases. Inquiries, concerns, and rumors identified during social listening call for rapid communication responses. These findings demonstrate a need for effective emergency risk communication strategies during a public health crisis that are informed by real-time evidence derived from ongoing social listening and tailored to local social and epidemic contexts. These strategies should not only meet immediate public information needs, but also debunk rumors and misinformation as they emerge.

Our data showed how local epidemic situations influenced public attitudes toward COVID-19 testing and highlighted the challenges facing governments when weighing the balance between epidemic control and socioeconomical livelihoods. This study was performed when the United Kingdom was under its first nationwide lockdown, while China had resumed complete normalcy since late March 2020. Tweets from London and the United Kingdom showed overwhelming support for both strategies, whereas more Weibo users expressed negotiated or oppositional positions of mass community-wide testing. In the United Kingdom, the government has long been criticized for being underprepared for the COVID-19 pandemic, including lacking testing capacity for both the general public and frontline workers [9,35]. Without reliable test results, very limited data were available to develop and introduce an exit strategy for the general lockdown, as health experts had no evidence to inform their decisions. After its implementation, the NHS Test and Trace program was widely criticized over the lack of convenience (ie, pricing and accessibility) and the exacerbation of COVID-19 inequalities, resulting in a new campaign being launched on July 30, 2020 [36] to encourage everyone with symptoms to undergo free testing. In China, comprehensive testing requirements around domestic travel have been in place since March 2020, when the country lifted its nationwide lockdown. Between June 12 and 22, 2020, the Beijing government led mass community-wide testing, with 2.95 million tests completed in 10 days due to a small resurgence of cases. Despite high perceived risk toward COVID-19, some Chinese residents questioned the overall cost-effectiveness of implementing a massive measure against such few cases. Public attitudes and sentiment constantly evolve with local epidemic situations, and as such, public communication about health risks and countermeasures must leverage real-time social listening and disease surveillance data to keep up.

There are geographic differences in public attitudes toward mass community-wide COVID-19 testing, with more people in London and Beijing, and adjacent areas opposing testing. In China, strong evidence indicates gender differences in attitudes toward mass community-wide COVID-19 testing, with more female residents supporting government-led testing. Consistent with the audience reception theory, our data showed that the public is a diverse heterogeneous set of people with varying experiences and needs. They access, process, and react to messages differently based on their individual backgrounds and views. Tailoring engagement strategies to the target community will be critical in increasing acceptance toward COVID-19 testing and other containment measures.

### Limitations

This study has some limitations. First, there is an inherent bias shared among all studies that utilize social media data, where users might present themselves differently online (eg, inflated perception) and/or represent a skewed younger population [37]. Nevertheless, findings from this study had very limited influence by curated perceptions as the investigation mainly focused on how aggregated social media data constituted a dynamic digital environment regarding COVID-19 testing, and how such an information environment affected individuals’ acceptance of control measures during a pandemic. Moreover, this study captured routine data from populations that may not be represented in traditional research designs. The opinions gathered via social listening could be less biased than those derived from traditional research methods, such as surveys and interviews, where unintended errors could be introduced by how questionnaires were presented and implemented (eg, reporting bias and acquiescence bias). Second, we were unable to extract demographic data from all Twitter and some Weibo profiles due to privacy restrictions, and the authenticity of the retrieved data was not directly verifiable. We therefore conducted account analyses of available Weibo profile data to assess data representativeness, which indicated a satisfactory level of comparability to the data in the official Weibo report. Third, data were downloaded daily to avoid the possible interference of comment removal by authorities. Lastly, the findings from this study are mostly exploratory and might not be generalizable due to the small sample size of posts reviewed (approximately 10%). A further investigation employing machine learning algorithms for big data analysis is needed.

### Conclusion

Policy makers tackling factors of COVID-19 testing hesitancy should focus on complacency, confidence, convenience, and communication in relation to testing. There is a need for more comparative studies to identify differences and similarities across populations and experiences with COVID-19 testing. Future infodemiology studies should integrate public and epidemic data (eg, traditional media, social media, polls, and disease surveillance data), both online and offline, and employ machine learning to enable rapid real-time analysis of big data for epidemic preparedness and response.

## Appendix

Multimedia Appendix 1 Workflow of the inclusion and exclusion process of tweets and Weibo posts.

Multimedia Appendix 2 Account analysis of COVID-19 test posts on social media.

Multimedia Appendix 3 Coding framework for COVID-19 test posts on social media.

## Abbreviations

NHS: National Health System
