# Supplementary figures and images for: Public Attitudes and Factors of COVID-19 Testing Hesitancy in the United Kingdom and China: Comparative Infodemiology Study
Source: JMIR Infodemiology. 2021 Aug 27;1(1):e26895. doi: 10.2196/26895 (PMC8404307; doi:10.2196/26895)

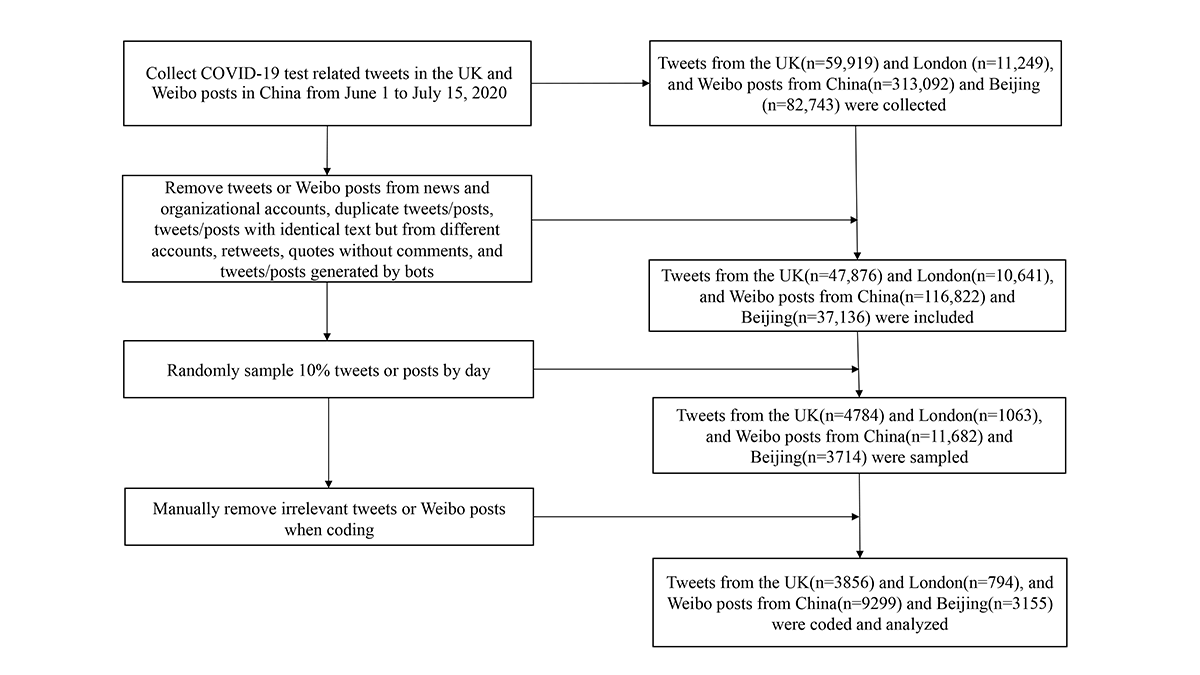

Supplement: Multimedia Appendix 1 [file infodemiology_v1i1e26895_app1.png]
