# Supplementary material for: Public Attitudes and Factors of COVID-19 Testing Hesitancy in the United Kingdom and China: Comparative Infodemiology Study
Source: JMIR Infodemiology. 2021 Aug 27;1(1):e26895. doi: 10.2196/26895 (PMC8404307; doi:10.2196/26895)
Supplement: Multimedia Appendix 2 [file infodemiology_v1i1e26895_app2.docx]

**Multimedia Appendix 2 Account analysis of COVID-19 test posts on social media, N (%)**

|  | **United Kingdom** | | | **China** | | |
| --- | --- | --- | --- | --- | --- | --- |
|  | **Total accounts** | **Post number per account** | **Total posts** | **Total accounts** | **Post number per account** | **Total posts** |
| Total | 3581 (92.9) |  | 3856 | 9067 (97.5) |  | 9299 |
| One post per account | 3361 (93.9) | 1 | 3361 | 8870 (97.8) | 1 | 8870 |
| Multiple posts per account | 220 (6.1) |  | 495 | 197 (2.2) |  | 429 |
| 2 posts per account | 183 | 2 | 366 | 170 | 2 | 340 |
| 3 posts per account | 26 | 3 | 78 | 22 | 3 | 66 |
| 4 posts per account | 6 | 4 | 24 | 4 | 4 | 16 |
| 5 posts per account | 3 | 5 | 15 |  |  |  |
| 6 posts per account | 2 | 6 | 12 |  |  |  |
| 7 posts per account |  |  |  | 1 | 7 | 7 |
