# Supplementary material for: Public Attitudes and Factors of COVID-19 Testing Hesitancy in the United Kingdom and China: Comparative Infodemiology Study
Source: JMIR Infodemiology. 2021 Aug 27;1(1):e26895. doi: 10.2196/26895 (PMC8404307; doi:10.2196/26895)
Supplement: Multimedia Appendix 3 [file infodemiology_v1i1e26895_app3.docx]

**Multimedia Appendix 3 Coding framework for COVID-19 test posts on social media**

| **Themes** | **Codes** | **Code definition** |
| --- | --- | --- |
| ***Uptake of COVID-19 tests*** | |  |
| 1 Self-reported uptake of COVID-19 tests | 1.1 Plan to take a test | Making plans (e.g., making an appointment) to take a COVID-19 test |
|  | 1.2 Have taken a test | Having taken a COVID-19 test or having obtained the test result. |
| 2 Driving force for taking a COVID-19 test | 2.1 Personal health needs | Taking a COVID-19 test due to personal worry and psychological comfort, exposure to COVID-19 risk, or self-symptoms. |
|  | 2.2 Mandatory testing policies for travel | Taking a COVID-19 test due to mandatory testing policies or regulations for travel, medical treatment, etc. |
|  | 2.3 Community-wide mass testing led by governments | Taking a COVID-19 test due to government-led community mobilization, organizing of work units, etc. |
| 3 Others taking COVID-19 tests |  | Family, friends, or colleagues plan to take a COVID-19 test or have taken the test. |
| ***Attitude towards COVID-19 tests*** | |  |
| 4 Attitude towards individual COVID-19 tests | 4.1 Dominant | Understanding, supporting, accepting individual COVID-19 tests |
|  | 4.2 Negotiated | Neutral, or mixed/cannot judge their attitudes. |
|  | 4.3 Oppositional | Rejecting (or doubting the necessity of) individual COVID-19 tests (opposite to the dominant position e.g., distrust, worry incidental risks, unwilling to take a test). |
| 5 Attitude towards mandatory testing policies for travel | 5.1 Dominant | Understanding, supporting, accepting mandatory testing policies or regulations for travel, medical treatment, etc. |
|  | 5.2 Negotiated | Neutral, or mixed/cannot judge their attitudes. |
|  | 5.3 Oppositional | Rejecting (or doubting the necessity of) the mandatory testing policies or regulations for travel, medical treatment, etc. (opposite to the dominant position) |
| 6 Attitude towards community-wide COVID-19 tests | 6.1 Dominant | Understanding, supporting, accepting mass COVID-19 testing |
|  | 6.2 Oppositional | Rejecting (or doubting the necessity of) mass COVID-19 testing (opposite to the dominant position). |
| ***Confidence: trust in COVID-19 tests*** | |  |
| 7 Concern on effectiveness of COVID-19 tests | 7.1 Trust tests to be effective | Trusting the test results to be effective, reliable, or accurate. |
|  | 7.2 Doubt effectiveness of tests | Distrusting or doubting the effectiveness, reliability, or accuracy of test results. |
| 8 Expiration date of COVID-19 tests |  | Mentioning expiration date (7 days) of COVID-19 tests. |
| 9 Incidental risks due to COVID-19 tests |  | Discussions on cross-infection risks and threats of asymptomatic carriers in a crowd gathering, etc. |
| ***Complacency: perception of COVID-19 risk*** | |  |
| 10 Perception of COVID-19 risk | 10.1 High risk | COVID-19 is susceptible and very severe if contracted, or COVID-19 epidemic is severe and fearful. |
|  | 10.2 Low risk | COVID-19 is not susceptible or not severe if contracted, or COVID-19 epidemic is not severe and there is nothing to fear. |
| ***Convenience: access to and experience with COVID-19 tests*** | |  |
| 11 Experience of taking a COVID-19 test | 11.1 Feel uncomfortable | Feeling uncomfortable or sick while taking a COVID-19 test. |
|  | 11.2 Feel nervous | Feeling nervous or afraid before taking a COVID-19 test. |
|  | 11.3 Do not feel uncomfortable | Not feeling uncomfortable or sick while taking a COVID-19 test. |
| 12 Logistical processes of obtaining a COVID-19 test | 12.1 Access to an appointment | It is difficult or slow to access to an appointment to take a COVID-19 test; it is easy or quick to access to an appointment to take a test somewhere. |
|  | 12.2 Wait time to take a test | Problems or improvement in arrangement of COVID-19 testing sites, including slow or quick queue, insufficient or sufficient medical staff, etc. |
|  | 12.3 Wait time for the test result | It is slow to get the COVID-19 test result; it is quick to get the test result somewhere. |
|  | 12.4 Others | Other aspects of obstacles or improvements in logistical process of obtaining a COVID-19 test. |
| 13 Tribute to medical staff |  | Paying tribute or showing respect to hard-working medical staff |
| 14 Price of COVID-19 testing |  | Mentioning or discussing the individual cost of COVID-19 testing. |
| 15 Priority groups for COVID-19 testing |  | Mentioning priority groups for COVID-19 testing, such as taxi drivers, and couriers, etc. |
| ***Communication: information inquiries and rumours related to COVID-19 tests*** | |  |
| 16 Information inquiries about COVID-19 tests |  | Questions about COVID-19 tests (e.g., how many people have received a test). |
| 17 Rumors about COVID-19 tests |  | Unproven expositions about or interpretations of COVID-19 testing related news, events, or problems that are of public interest (i.e. rumors, fake news, or misinformation, etc.) |
| **18 Doubt the governmental practices** |  | Expressing doubt or dissatisfaction with the governmental practices or policies, such as taxes for COVID-19 testing, untimely publication of data, etc. |
| **19 Others** |  | Expressing other views on COVID-19 tests and cannot be classified to the above categories. |
| **20 Irrelevance** |  | Posts without personal opinions (Including News, posts from officials and organizations, quoted without comments, etc), or personal opinions that are irrelevant to the research topic. |
